# Symptomatic partial anomalous pulmonary venous connection in a kitten

**DOI:** 10.1111/jvim.15934

**Published:** 2020-10-16

**Authors:** Tung Hsueh, Chung‐Chun Yang, Shiun‐Long Lin, I‐Ping Chan

**Affiliations:** ^1^ Veterinary Medical Teaching Hospital, National Chung Hsing University Taichung Taiwan; ^2^ Department of Veterinary Medicine College of Veterinary Medicine, National Chung Hsing University Taichung Taiwan

**Keywords:** agitated saline contrast imaging, computed tomography angiography, congenital cardiovascular disease, feline, heart failure

## Abstract

A 3‐month‐old intact female American Shorthair cat, with syncope and tachypnea, underwent cardiac examination which identified no heart murmur or gallop. Thoracic radiography disclosed mild generalized enlargement of the cardiac silhouette and a bronchial and interstitial pattern throughout the lungs. Echocardiography identified tubular structures near the left atrium. After agitated saline contrast imaging, persistent left cranial vena cava with unroofed coronary sinus was suspected. Computed tomography angiography showed the right cranial, right caudal and left caudal pulmonary veins draining into the coronary sinus and flowing into the right atrium. The left cranial pulmonary vein drained normally into the left atrium. Partial anomalous pulmonary venous connection (PAPVC) was diagnosed. The kitten was treated with diuretics but died of heart failure 2 months later. Permission for necropsy was not granted. This case represents symptomatic PAPVC in a kitten. Most pulmonary veins were connected abnormally with the coronary sinus. The prognosis was grave because of refractory heart failure.

**Abbreviations**
APVC: anomalous pulmonary venous connection
CS: coronary sinus
CTA: computed tomography angiography
PAPVC: partial anomalous pulmonary venous connection
PFO: patent foramen ovale
PLCVC: persistent left cranial vena cava

## 1 INTRODUCTION

Partial anomalous pulmonary venous connection (PAPVC) is a rare congenital cardiovascular abnormality. In veterinary literature, anomalous pulmonary venous connection (APVC) or drainage has been reported in dogs,^1^, ^2^, ^3^, ^4^, ^5^, ^6^, ^7^ cats,^8^ a foal,^9^ and chickens.^10^ In fetal life, the primitive pulmonary veins from the lung buds develop from the splanchnic plexus, which communicates with the systemic venous system, and connects to the left atrium. As development proceeds, the connection between pulmonary veins and the systemic venous system disappears. If the communication between pulmonary veins and the systemic venous system persists, total APVC or PAPVC would be diagnosed depending on the degree of persistent connections.^11^

## 2 CASE PRESENTATION

A 3‐month‐old intact female American shorthair cat was admitted to the Veterinary Medical Teaching Hospital of the National Chung‐Hsing University for cardiac examination. An episode of syncope was observed 1 month earlier, which was followed by 2 weeks of tachypnea. The kitten was hospitalized for supportive care at a local animal hospital and the referring veterinarian suspected congestive heart failure secondary to congenital heart disease.

**FIGURE 1:**
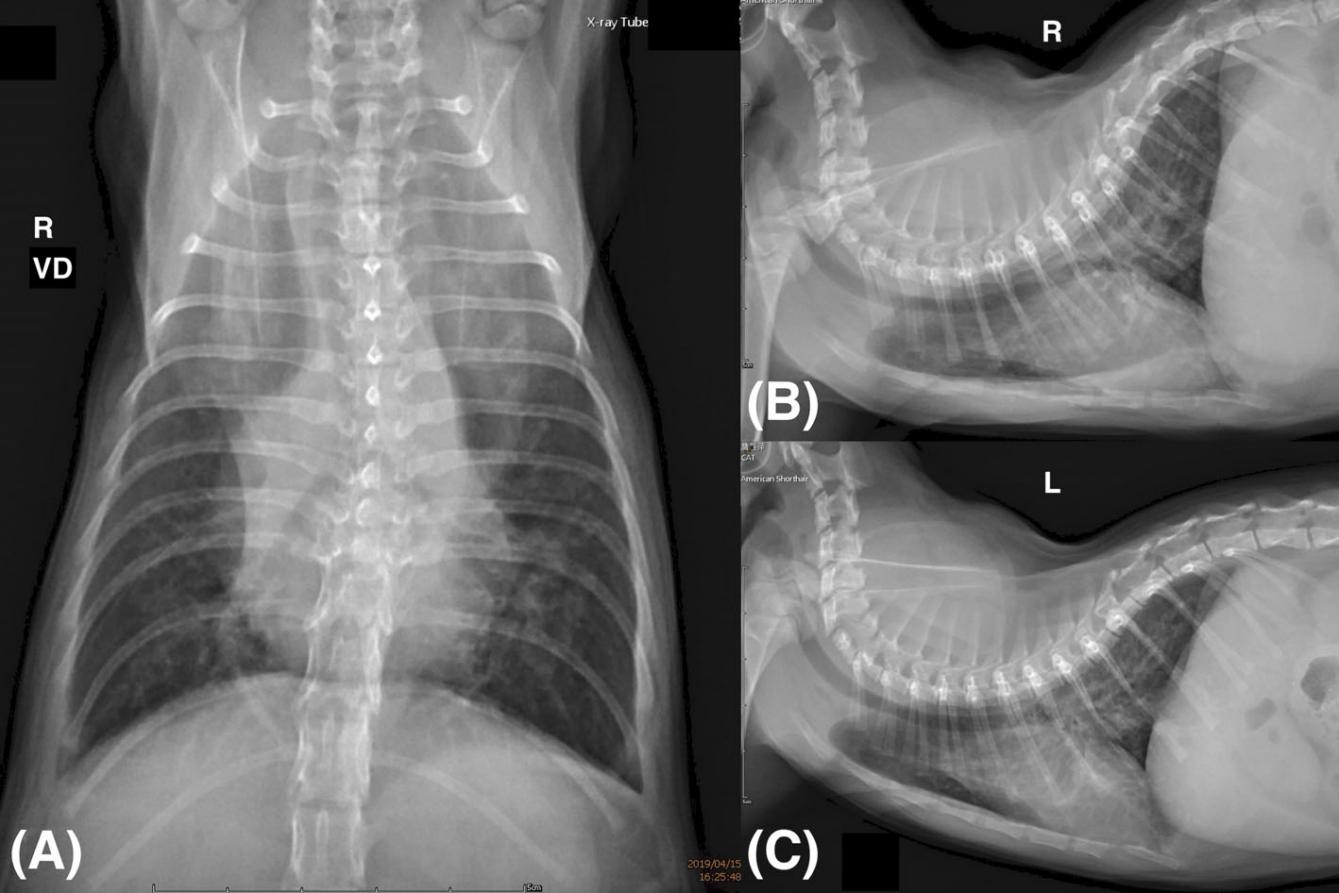
Thoracic radiography after admission. Diffuse bronchial and interstitial pattern on the A, ventral‐dorsal; B, right recumbent; and C, left recumbent views

On presentation, the kitten was bright, alert, and responsive, and weighed 1.45 kg with mild muscle loss. Thoracic auscultation was normal, and the heart rate was 216 beats per minute. The kitten was sedated using 0.3 mg/kg butorphanol IM (Butomidor, richter pharma AG, Wels, Oberösterreich, Austria) combined with 0.003 mg/kg dexmedetomidine (DEXDOMITOR, Zoetis, Parsippany, New Jersey) to minimize stress and facilitate evaluation. Thoracic radiography disclosed generalized cardiomegaly with a vertebral heart score of 8.3 (normal, 7.3 ± 0.49),^12^ and a diffuse bronchial interstitial pattern combined with pulmonary overcirculation (Figure 1).

**FIGURE 2:**
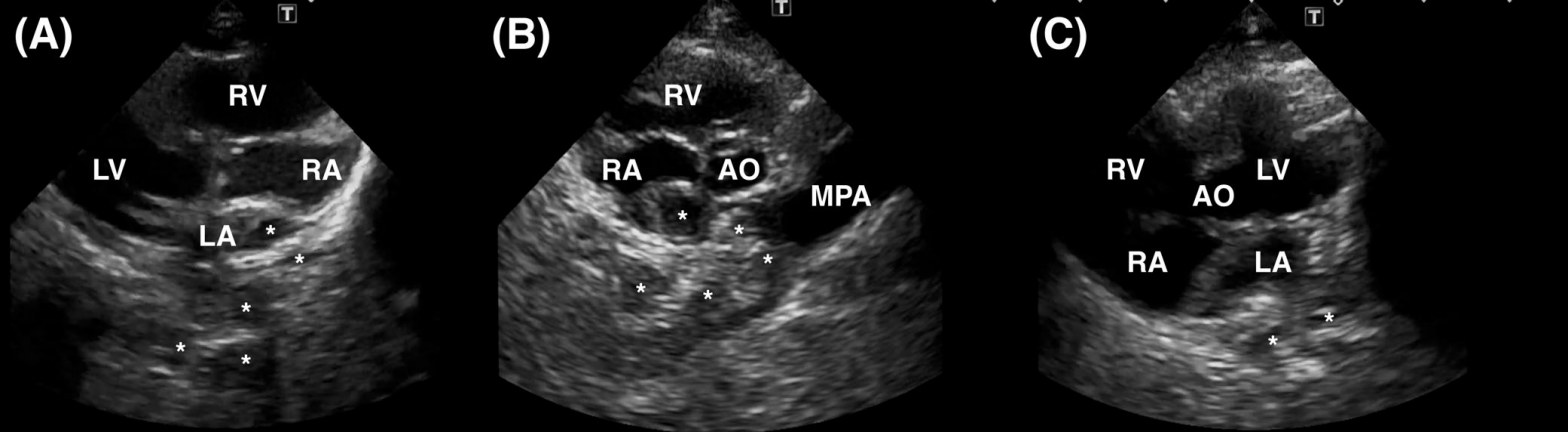
Enlargement of the right atrium, ventricle, and main pulmonary artery on the right parasternal long‐axis view (A), the right short‐axis view at the heart base level (B), and the left apical long‐axis view (C). In these views, the anomalous tubular structures near the left and right atria were marked with asterisks. AO, aorta; LA, left atrium; LV, left ventricle; MPA, main pulmonary artery; RA, right atrium; RV, right ventricle

Echocardiography was performed under sedation, and showed dilatation of the right atrium, right ventricle, and main pulmonary artery (Figure 2). Moderate tricuspid regurgitation^13^ was noted, with an average velocity of 2.89 m/s. These findings were consistent with mild pulmonary hypertension. The ratio of pulmonary and systemic blood flow (Qp/Qs) estimated by 2‐dimensional echocardiography and pulsed‐wave Doppler interrogation was 3.0, based on stroke volume. The Qp/Qs was 3.21 if heart rate per minute was taken into consideration, further confirming the presence of pulmonary overcirculation. Left heart‐related measurements, including dimensions and volumes, were within normal ranges, and color Doppler interrogation identified moderate mitral regurgitation. A tethered anterior leaflet potentially indicated mitral valve dysplasia. In both the short‐ and long‐axis views, tubular structures were visible near the left atrium (Figure 2). Persistent left cranial vena cava (PLCVC) was the first differential diagnosis considered. Hence, IV catheters were placed in the cephalic veins of both forelimbs to perform agitated saline contrast imaging. Microbubbles entered the pulmonary artery through the right atrium and right ventricle and were detained in these sites via the right cephalic vein for a few seconds. Microbubbles injected from the left cephalic vein were present in the right atrium. Additionally, microbubbles appeared in 1 of the tubular structures and the left atrium simultaneously. Based on these echocardiographic findings, we tentatively diagnosed PLCVC with unroofed coronary sinus (CS). To confirm this, we investigated the underlying cause of pulmonary hypertension and evaluated the possibility of treatment by scheduling computed tomography angiography for 3 weeks later (CTA; Brilliance iCT 256 slice, Philips, Eindhoven, The Netherlands). Medications administered PO included furosemide (4.3 mg/kg PO q12h), pimobendan (0.27 mg/kg PO q12h), benazepril (0.32 mg/kg PO q12h), and spironolactone (1.6 mg/kg PO q12h). The dosage of furosemide was kept the same as in the original prescription provided by the referring veterinarian, because there was no evidence of active heart failure at presentation to our hospital.

**FIGURE 3:**
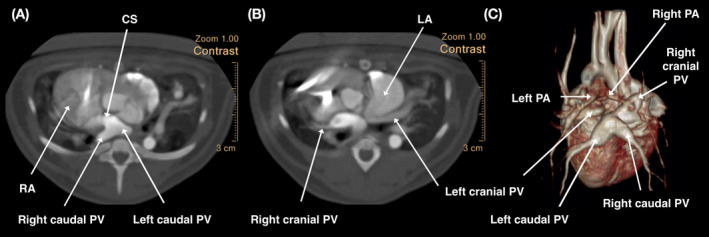
Blood with contrast from the right caudal pulmonary veins and left caudal pulmonary vein drained into the right atrium and then into the left atrium (A). The left cranial pulmonary vein connected to the left atrium (B). Dorsal aspect of the reconstructed heart model (C), a large venous structure is observed. CS, coronary sinus; RA, right atrium; RV, right ventricle; PV, pulmonary vein; PA, pulmonary artery

On the day of CTA, the kitten was sedated using butorphanol (0.3 mg/kg IM) for peripheral venous catheter placement, combined with 0.003 mg/kg dexmedetomidine to slow the heart rate during the procedure. Contrast medium (Iopamiro 370, Bracco Imaging, Milan, Italy) was administered using an injector (MEDRAD Stellant CT Injection System, Medrad, Pennsylvania) into the left cephalic vein. The right cranial, right caudal, and left caudal pulmonary venous branches were merged into a large venous sinus located in the dorsal aspect of the heart between the right and left atria. The blood flow of this venous sinus, therefore, drained into the right atrium through the CS rather than into the left atrium; contrast media entered the left atrium later (Figure 3A). The left cranial pulmonary vein was not connected with other pulmonary veins and drained normally into the left atrium (Figure 3B). The 3‐dimensional reconstruction image of both the heart and large vessels is shown in Figure 3C. These findings suggested a diagnosis of PAPVC with patent foramen ovale (PFO). Slightly low oxygen saturation (SpO_2_) between 87 and 88% was later identified. The clinical manifestations of both pulmonary overcirculation and mildly decreased systemic oxygenation were indicative of non‐obstructive APVC.

Treatment is to surgically reconstruct the pulmonary veins, which is accomplished by cardiopulmonary bypass, but cardiopulmonary bypass was not possible in a kitten weighing only 1.45 kg because of small blood volume, risks of electrolyte disturbance, hemodilution, and limitation of small vessel diameter for cannulation.^14^ After discussion with the owner, cardiac medications including furosemide (4.3 mg/kg PO q12h), pimobendan (0.25 mg/kg PO q12h), benazepril (0.32 mg/kg PO q12h), and spironolactone (1.6 mg/kg PO q12h) were prescribed to control heart failure and maintain quality of life. Two months after diagnosis, the referring veterinarian reported that the kitten had died of heart failure; permission for necropsy was denied.

## 3 DISCUSSION

This case report describes PAPVC, a rare congenital cardiovascular abnormality, in a kitten. Descriptions for this type of abnormality include the terms “connection” and “drainage.” The term “connection” indicates an anomalous venoatrial connection, whereas the word “drainage” describes the concept of abnormal pulmonary venous return, despite normal anatomical connection.^15^ Depending on the diagnostic evaluation performed, “connection” is the preferred description in this case.

Tubular structures, seen in both long‐ and short‐axis echocardiographic views, were indicative of PLCVC, which also is a rare congenital cardiovascular abnormality in cats, but it is more common in dogs. Furthermore, microbubbles injected from the left cephalic vein were visible in the tubular structure and then appeared in the left atrium. However, PLCVC alone cannot explain why microbubbles appeared in the left atrium, because PLCVC in 90% of cases in humans is connected to the CS and eventually drains into the right atrium.^16^ Unroofed CS is another developmental disorder that frequently is identified together with PLCVC and was considered a concomitant abnormality. The CS collects blood supplied from the myocardium and serves as the final portion of coronary venous circulation. It is transversely located in the left atrioventricular groove on the dorsal aspect of the heart. Unroofed CS represents either single or multiple defects in the shared wall between the CS and the left atrium, allowing blood communication between the right and left atria through the incomplete CS. Therefore, it is considered a type of atrial septal defect.^17^ Before advanced imaging, a diagnosis of PLCVC combined with unroofed CS seemed likely in this case.

Interestingly, the final diagnosis was PAPVC after CTA. The PAPVC also can account for the echocardiographic findings, because the tubular structures surrounding the heart base were a dilated CS and anomalous pulmonary veins. Microbubbles injected from either the right or left cephalic vein entered the right atrium first and then the left atrium through the PFO. The right atrium received higher than normal blood volume (systemic and most pulmonary venous return), with CS dilatation, with evidence of microbubbles arriving simultaneously. In the setting of PAPVC with PFO rather than PLCVC with an unroofed CS, no discrepancy is expected between the results of agitated saline contrast, which was injected from the right and left cephalic vein. Microbubbles injected from peripheral veins arrived at the right atrium together with venous return, and the fluctuating right atrial pressure in different cardiac cycles and respiratory phases could result in an intermittent appearance of microbubbles in the CS and left atrium via the PFO, which might explain why we found different results between the 2 microbubble tests.

Partial anomalous pulmonary venous connection refers to ≥1, but not all, pulmonary veins being connected to the systemic venous circulation rather than the left atrium. In contrast to total APVC, most humans with PAPVC are asymptomatic and the condition typically remains undiagnosed until adulthood.^18^, ^19^ Patients may not show any clinical signs or may develop dyspnea with exertion, or even show signs of cyanosis and exercise intolerance. Both the timing of diagnosis and the severity of symptoms depend on the number of affected pulmonary veins because patients with ≥1 anomalous pulmonary vein could be diagnosed earlier because of severe clinical signs, compared to patients with only 1 abnormal pulmonary vein.^20^ In this case, the right cranial, right caudal, and left caudal pulmonary veins drained to the CS. The left cranial pulmonary vein was the only 1 that developed normally, causing considerable left‐to‐right shunting. Long‐term lung overcirculation is a potential cause of both pulmonary hypertension and pulmonary edema, which were observed in this kitten, and result in syncope and tachypnea. These signs are consistent with the findings of non‐obstructive APVC, indicating that patients may present with both mildly decreased systemic desaturation and congestive heart failure, resulting from increased hydrostatic pressure caused by pulmonary overcirculation.^21^ The estimated pulmonary and systemic flow (Qp/Qs) in our patient was approximately 3. In human patients, surgical repair is recommended if the shunt ratio is found to be hemodynamically important (Qp/Qs > 1.5) because such patients are at a higher risk of pulmonary hypertension and heart failure.^22^ These clinical findings are similar to total APVC, another congenital venoatrial developmental abnormality that shares similar embryogenesis, with all pulmonary veins being abnormally connected. Total APVC can be classified into 4 types depending on location.^20^ The anomaly in our kitten closely resembled cardiac type total APVC, in which the pulmonary veins drain into the right atrium directly or through the CS.

A previous case report described a cat with PAPVC and suspected pulmonary hypertension.^8^ In contrast to our case, the cat of the previous report was asymptomatic at 14 months of age and was referred for evaluation of a heart murmur that was heard incidentally. Computed tomography identified that pulmonary veins from the caudal lung lobes drained into the caudal vena cava with bilateral cranial pulmonary veins that were connected normally to the left atrium. No structural cardiac disease that could result in the left parasternal systolic murmur could be identified. By comparing these 2 cases, it seems that PAPVC could be tolerated, and clinical manifestations and prognosis could vary depending on the extent of abnormal pulmonary veins.

During fetal life, the foramen ovale is a route by which blood can bypass the uninflated lungs, and it should close after birth because of decreased right‐sided pressure. As the right‐sided heart pressure and volume remain high after birth, the foramen ovale cannot close, and still allows communication between the right and left atria, a condition known as PFO, which permits some mixture of oxygenated and deoxygenated blood to enter the systemic circulation. In our case, the presence of PFO could be indirectly identified by the slightly lower SpO_2_, because no other shunting was observed. Moreover, contrast material entered the left atrium after the right atrium, also supporting the presence of a PFO.

In conclusion, we described symptomatic PAPVC in a kitten. The clinical manifestation of this rare congenital cardiovascular disease can vary depending on the individual. In veterinary patients, conventional radiography and echocardiography are supportive for PAPVC diagnosis, whereas advanced diagnostic imaging, such as CTA, that provides 3‐dimensional images can improve understanding. Future reports, including studies before and after death, could provide more information and advance knowledge of this disease.

## CONFLICT OF INTEREST DECLARATION

Authors declare no conflict of interest.

## OFF‐LABEL ANTIMICROBIAL DECLARATION

Authors declare no off‐label use of antimicrobials.

## INSTITUTIONAL ANIMAL CARE AND USE COMMITTEE (IACUC) OR OTHER APPROVAL DECLARATION

Authors declare no IACUC or other approval was needed.

## HUMAN ETHICS APPROVAL DECLARATION

Authors declare human ethics approval was not needed for this.
